# Point Defect Detection and Classification in MoS_2_ Scanning Tunneling Microscopy Images: A Deep Learning Approach

**DOI:** 10.3390/molecules30122644

**Published:** 2025-06-18

**Authors:** Shiru Wu, Guoyang Chen, Si Shen, Jiaxu Yan

**Affiliations:** 1 School of Arts and Sciences, Shanghai Dianji University, Shanghai 200245, China; srwu@njtech.edu.cn; 2 University of Chinese Academy of Sciences, Chinese Academy of Sciences, Beijing 100049, China; chenguoyang21@mails.ucas.ac.cn; 3 State Key Laboratory of Luminescence Science and Technology, Changchun Institute of Optics, Fine Mechanics and Physics, Chinese Academy of Sciences, Changchun 130033, China

**Keywords:** MoS_2_, scanning tunneling microscopy, deep learning, defect detection, convolutional neural network

## Abstract

Point defects in two-dimensional materials such as MoS_2_ can critically impact their electronic and optoelectronic properties. Precise identification of these defects is essential for understanding defect physics and device performance. In this work, we acquire high-resolution scanning tunneling microscopy (STM) images of monolayer MoS_2_ and apply the Segment Anything Model (SAM) to automatically segment possible defect regions in the STM images. Each segmented region is then classified by a convolutional neural network (CNN) architecture into defect categories. This deep learning pipeline is trained on augmented STM image data and evaluated against manual annotations. The model achieves a classification accuracy of 95.06% on a modest dataset comprising merely 198 samples, demonstrating its robustness despite limited data availability. We also perform density functional theory (DFT) calculations of representative defect structures to support interpretation of the STM features. Charge density isosurfaces of the DFT models reveal localized mid-gap states associated with sulfur vacancies, consistent with STM observations. The integration of SAM segmentation, CNN classification, and DFT modeling provides a comprehensive approach to quantify defect populations in MoS_2_. These results show the potential of combining data-driven image analysis with physics-based modeling to accelerate defect characterization in 2D materials.

## 1. Introduction

Atomically thin transition metal dichalcogenides (TMDs), represented by MoS_2_, are promising semiconductor materials for next-generation devices [1,2], due to their attractive properties such as high carrier mobility [3], direct bandgap [4], valley polarization [5], and piezoelectricity [6]. Intrinsic point defects in TMDs have received significant research interest because they inevitably exist and strongly influence the physical properties of the host material. With the physical confinement and reduced screening in monolayer TMDs, point defects can act as efficient traps for free electrons, holes, or excitons. Therefore, they are often considered responsible for device-to-device variations in transport curves [7,8] or trionic photoluminescent (PL) emission [9]. On the other hand, specific types of defects, such as chalcogen monovacancies, exhibit unique properties like giant spin–orbit splitting [10] and subgap excitonic states [11], which can serve as magnetic impurities [12], local spin qubits [13], or single-photon emitters [14,15] in advanced quantum devices. Scanning tunneling microscopy (STM) provides atomic-resolution images of the MoS_2_ surface, resolving individual defects and their associated electronic contrast [10,16,17,18,19,20,21]. However, manual identification and counting of defects in large datasets of STM images is laborious and subjective [16,17,22]. Recent work has demonstrated the application of convolutional neural networks (CNNs) and other deep learning (DL) methods to automate defect detection in 2D materials. For instance, an ensemble of U-Net CNNs was used to detect atomic-scale defects in STM images of WSe_2_, achieving an F_1_ score of ∼0.66 and even generalizing to related materials [23]. Similarly, Chen et al. developed a deep learning framework for MoS_2_ STM images using data augmentation and noise filtering, reporting *F*-score ≈ 0.86 for defect detection [24]. These studies highlight the power of CNNs in learning abstract features from complex STM data. Beyond CNN classification, recent advances in vision foundation models have introduced powerful segmentation tools for microscopy. The Segment Anything Model (SAM) has been adapted for cell and organelle segmentation [25], and it can be used to isolate individual objects or regions in STM images with minimal annotation. By coupling SAM segmentation with a CNN classifier, one can first identify candidate defect regions and then determine their type. In this work, we integrate SAM-based segmentation with a CNN classifier to automate detection and classification of point defects in MoS_2_ STM images. We also situate this approach within the theoretical framework of defect physics. Density functional theory (DFT) calculations of MoS_2_ with point defects provide insight into their atomic and electronic structure. Prior DFT studies confirm that sulfur vacancies have low formation energy and produce localized mid-gap charge density [26]. By computing charge density isosurfaces and local density of states for vacancy models, we connect the CNN-based image classification to underlying physics. Our study combines experimental STM, DFT modeling, and state-of-the-art deep learning to achieve accurate, high-throughput defect analysis, advancing the use of machine learning in materials characterization [21,27,28,29,30,31,32].

## 2. Results and Discussion

**Figure 1:**
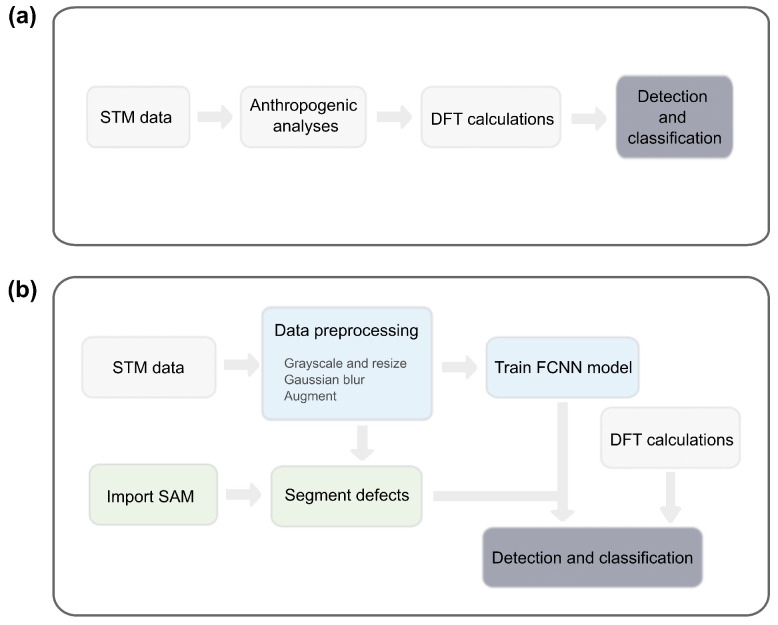
The framework for defect detection and classification. (**a**) The traditional workflow. (**b**) The ML method workflow.

The detection and classification of MoS_2_ point defects in STM images that combines STM experiments with machine learning techniques, as illustrated in Figure 1.

**Figure 2:**
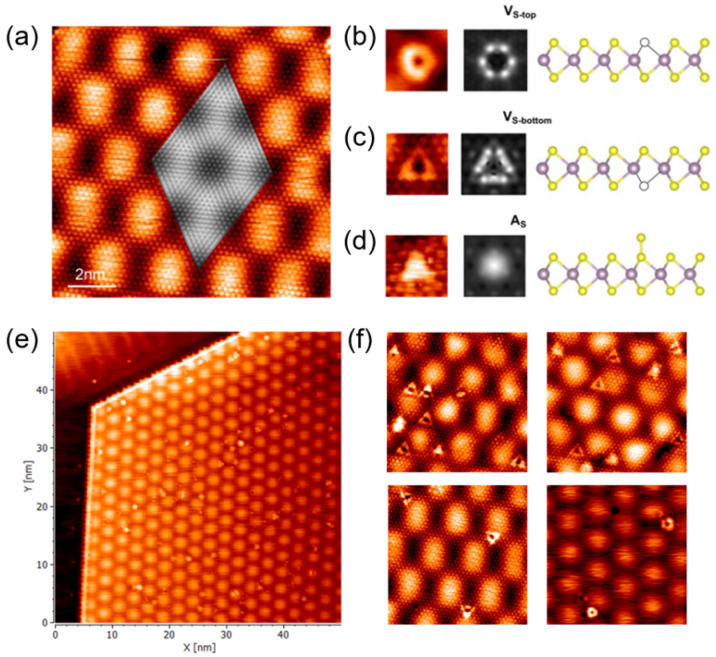
(**a**) The experimental (decorated) and simulated (grey) STM images. (**b**) The V_*S*−*top*_ defect images and structural model. (**c**) The V_*S*−*bottom*_ defect images and structural model. (**d**) The A_*S*_ defect images and structural model. From left to right, the experimental defect image, the simulated defect image, and the schematic structure of the defect are shown. (**e**) Experimental STM images of a single isolated monolayer ${\mathrm{MoS}}_{2}$ island (∼40–50 nm), clearly showing defects within the film. (**f**) STM images highlighting various regions with point defects.

Figure 2a presents a filled-state scanning tunneling microscopy (STM) image of monolayer ${\mathrm{MoS}}_{2}$ on Au(111), clearly exhibiting the ${\mathrm{MoS}}_{2}$ atomic lattice overlaid by a distinct Moiré superstructure with a periodicity of approximately 3.27 nm, arising from lattice mismatch. Figure 2e,f are experimental STM images of monolayer ${\mathrm{MoS}}_{2}$ island, clearly showing various point defects within the film. To systematically identify defects, experimental images are compared with constant-current Tersoff–Hamann simulations (bias voltage: −1.0 V) of three distinct defect models: top-layer sulfur vacancy ($V_{S}^{\mathrm{top}}$), bottom-layer sulfur vacancy ($V_{S}^{\mathrm{bot}}$), and sulfur interstitial ($A_{S}$). Simulation results reveal unique signatures for each defect type: the $V_{S}^{\mathrm{top}}$ defect exhibits a hexagonally symmetric halo around the vacancy, while the $V_{S}^{\mathrm{bot}}$ defect manifests as a triangular dark depression bordered by bright lobes at its vertices. These simulated features closely match experimental observations; thus, defects displaying the characteristic hexagonal halo (Figure 2a) are conclusively identified as top-layer sulfur vacancies, whereas triangular depressions correspond to bottom-layer sulfur vacancies. Additionally, the $A_{S}$ interstitial appears consistently as a localized bright spot centered within a Mo hexagon, observed clearly in both simulations and experiments. The strong correspondence in shape, symmetry, and intensity between simulated and experimental STM images at −1.0 V bias conclusively supports these defect assignments, directly reflecting local electronic redistributions associated with each defect structure.

**Figure 3:**
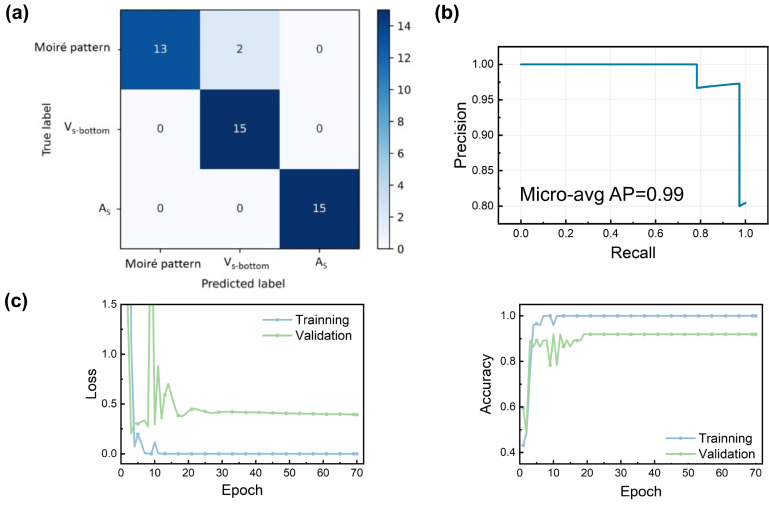
(**a**) Confusion matrix. (**b**) Micro-average PR curves. (**c**) The loss and accuracy of the training set and validation set.

To effectively train our CNN model using supervised learning, careful preprocessing of 150 experimental STM images was conducted. Accurate machine learning models typically require extensive datasets of high-resolution images. However, acquiring large-scale, high-quality STM images is resource-intensive, resulting in limited datasets often affected by noise and boundary artifacts. To mitigate these limitations, we rigorously filtered the original STM dataset, selecting only images with clearly defined defect boundaries, classifying them into three distinct categories: two recognized defects ($A_{S}$ and $V_{S}^{\mathrm{bot}}$) and darker Moiré regions resulting from ${\mathrm{MoS}}_{2}$–Au(111) interactions. During preprocessing, data augmentation techniques—including random cropping, rotation, scaling, and horizontal/vertical flipping—expanded the original set of 150 images to 198 (comprising 30 Moiré patterns, 118 $V_{S}^{\mathrm{bot}}$ defects, and 50 $A_{S}$ defects). Although augmentation does not generate fundamentally new information, it enhances feature relevance, reduces background variability, and significantly improves the generalization capability of the CNN model. The augmented dataset was divided into training and validation subsets using a 4:1 ratio. Additional preprocessing steps included grayscale conversion, resizing images to 256×256 pixels, Gaussian blurring to suppress noise, Fast Fourier Transform (FFT)-based filtering to remove high-frequency noise, inverse FFT application, and contrast adjustment. These preprocessed images were then utilized for training the CNN model. The model’s efficacy was rigorously assessed via five-fold cross-validation, achieving a commendable accuracy of 95.06%. Further model testing involved evaluating 15 previously unseen images per defect class. The resulting confusion matrix (Figure 3a) indicates excellent discrimination capability among $V_{S}^{\mathrm{bot}}$, $A_{S}$, and Moiré patterns. Furthermore, micro-average PR curves (Figure 3b) yielded an AP score of 0.99 on the validation set, confirming model reliability. Accuracy and loss curves (Figure 3c) further demonstrate robust convergence, highlighting the model’s efficiency and stability, particularly beneficial in scenarios constrained by limited training data.

**Figure 4:**
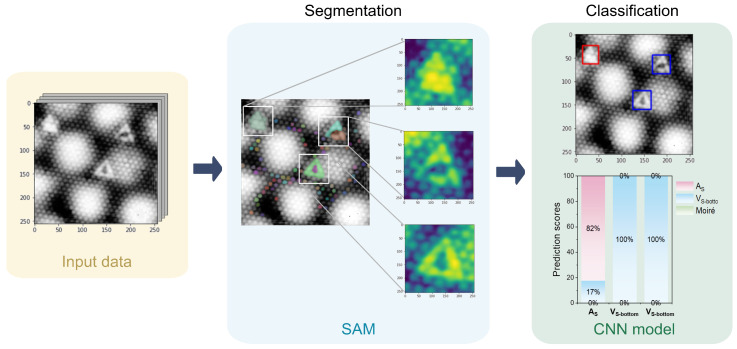
Defect detection and classification architecture results.

Defect detection in STM images of ${\mathrm{MoS}}_{2}$, especially at scales ranging between 10–15 nm, presents significant challenges due to intrinsic atomic lattice and Moiré pattern-induced noise interference. Conventional image recognition models such as Single Shot MultiBox Detector (SSD) and OpenCV-based methods demonstrate insufficient accuracy under these conditions [33,34]. To overcome this limitation, the Segment Anything Model (SAM) was integrated into our workflow to enhance defect detection capabilities. The defect detection process commenced with Gaussian convolution preprocessing (kernel size: 3×3), designed to blur atomic-scale features while preserving clear defect boundaries. Although the accuracy of SAM is inherently dependent on original image quality, this preprocessing step substantially improved defect detectability. Subsequent to preprocessing, SAM segments potential defect regions from the STM images. To further refine defect localization, bounding boxes generated by SAM were expanded outward by 7 pixels. These expanded regions were then processed by the CNN model, leveraging a softmax classifier to generate classification scores corresponding to different defect types. A defect was confirmed if the corresponding classification score exceeded 0.8. These scores were visualized as percentage-based stacked bar charts (Figure 4, classification module), facilitating straightforward interpretation. The integrated SAM–CNN approach yielded high detection accuracy for $V_{S}^{\mathrm{bot}}$ defects, outperforming the classification of $A_{S}$ defects. This discrepancy primarily stems from the dataset imbalance—118 images for $V_{S}^{\mathrm{bot}}$ versus only 50 images for $A_{S}$ defects. These findings underscore the critical importance of balanced datasets for optimal training efficacy. Future studies should explore enhanced data augmentation strategies or employ transfer learning to bolster classification accuracy for less-represented defect types such as $A_{S}$ defects.

## 3. Materials and Methods

### 3.1. Sample Preparation and STM Measurements

Monolayer MoS_2_ films were grown on Au(111) substrates using molecular beam epitaxy (MBE) under ultrahigh vacuum (UHV) conditions [35,36]. The Au(111) substrate was first cleaned through multiple cycles of sputtering and annealing to ensure a pristine surface. Molybdenum (Mo) was then deposited onto the Au(111) surface using an electron-beam evaporator. Subsequently, the chamber was backfilled with H_2_S gas at a partial pressure of $5\times 10^{-6}$ mbar while maintaining the substrate temperature at 200 °C for 40 min to facilitate the conversion of metallic Mo into small-domain MoS_2_. This process resulted in high-quality, defect-free MoS_2_ monolayers with a typical defect density of approximately $2\times 10^{11}$ cm^−2^, which is significantly lower than that of mechanically exfoliated MoS_2_ samples [17]. STM was employed to characterize the atomic structure and defects in the MoS_2_ monolayers. The STM measurements were performed at 4.5 K using electrochemically etched tungsten tips.

### 3.2. Data Preprocessing and Augmentation

Raw STM images often contain drift, tilt, and noise artifacts. Prior to analysis, all images were plane-corrected and flattened line-by-line to remove background slope. A Gaussian smoothing filter was applied to reduce high-frequency noise, and contrast-limited adaptive histogram equalization (CLAHE) was used to enhance feature visibility, as commonly performed in STM image analysis. After preprocessing, a set of clean STM images was manually annotated by marking defects (vacancies, impurities, etc.).

### 3.3. SAM Segmentation of Defects

To localize potential defects in each STM image, we applied the Segment Anything Model (SAM). SAM was used to generate segmentation masks for all salient features in the image, which is a foundational vision model for image segmentation tasks that achieves zero-shot generalization. It uses a hybrid architecture consisting of a ViT-based image encoder, a fast encoder for user-defined inputs (e.g., points, bounding boxes), and a lightweight mask decoder to generate high-precision segmentation masks without fine-tuning for specific tasks. SAM is trained on the SA-1B dataset (containing 11 million images and 1 billion masks), and demonstrates robust performance in different domains by separating object semantics from structural priors. From these masks, we selected candidate regions (e.g., bounded contours) that could correspond to point defects. Each region was expanded by a fixed margin to include local context. These segmented subimages (masks) were used as input for the classification CNN. By using SAM, we ensure that the CNN focuses on individual feature patches, improving robustness to varying backgrounds and image artifacts. This approach also allows processing of full-size STM images: SAM first isolates objects, and then the CNN classifies them.

### 3.4. Convolutional Neural Network Architecture

In this study, convolutional neural networks (CNNs) were used for image classification tasks. CNNs have successfully demonstrated excellent performance in tasks such as image recognition and target detection, providing a new methodological basis for high-throughput material characterization [37]. It is a deep learning architecture inspired by biological visual systems, designed to process high-dimensional data with local spatial correlation (such as images, spectra, and topological morphology), automatically extract local features of images (such as edges, textures, etc.) through multi-layer convolution operations, and fuse them layer by layer into high-level semantic information (such as object shape and structure), and finally achieve image classification through fully connected layers. The CNN model is implemented using the TensorFlow framework [38]. It automatically learns the progressive representation of data from low-level local patterns to high-level semantic abstraction through a hierarchical feature extraction mechanism. The network consists of three consecutive convolutional layers, each containing a learnable 3×3 convolution kernel and a ReLU activation function, followed by a pooling layer to downsample the feature map to reduce the number of parameters and enforce translation invariance. The final convolutional feature map is aggregated through a fully connected layer and mapped through a softmax classifier to derive a probability distribution over defect classes (e.g., sulfur vacancies, molybdenum vacancies, impurities, or background). During training, the model optimizes the categorical cross entropy loss using the Adam optimizer, and key hyperparameters (learning rate, batch size) are tuned on a validation set. In a representative configuration—70 epochs, a learning rate of $1\times 10^{-3}$, and a batch size of 32—training takes about 18 min on a GPU-accelerated workstation.

### 3.5. Training and Evaluation Metrics

The CNN was trained to classify segmented patches into specific defect categories or non-defective background through iterative optimization using training/validation sets, with final evaluation conducted on a held-out test set. Performance evaluation employs three core metrics: Accuracy quantifies global classification correctness through the ratio of correct predictions:

(1) $$\mathrm{Accuracy}=\frac{TP+TN}{TP+TN+FP+FN}$$

where TP (True Positives) denotes correctly identified defect samples, TN (True Negatives) represents correctly classified background regions, FP (False Positives) indicates background misclassified as defects, and FN (False Negatives) corresponds to undetected defects. Precision–Recall metrics provide complementary insights: Precision measures positive prediction reliability through the ratio Precision=TP/(TP+FP), while Recall evaluates positive class coverage via Recall=TP/(TP+FN), expressed, respectively, as:

(2) $$\mathrm{Precision}=\frac{TP}{TP+FP},\;\mathrm{Recall}=\frac{TP}{TP+FN}$$

Micro-Average PR curves extend these metrics to multi-class scenarios by aggregating predictions across all classes. This approach unifies multi-classification as binary subproblems, accumulates global TP/FP/FN counts, and computes unified precision/recall metrics to assess overall model performance. Confusion matrix analysis utilizes a table where rows show actual classes and columns show predicted classes. Each cell indicates how many samples of a true class were classified into each predicted class, highlighting model biases, common misclassifications, and per-class accuracy.

### 3.6. DFT Modeling of Defects

To confirm the experimentally observed defect types, we performed DFT calculations using the Vienna Ab Initio Simulation Package (VASP) [39]. We employed the projector-augmented wave (PAW) method and the Perdew–Burke–Ernzerhof (PBE) exchange-correlation functional, with the optB86b-vdW functional to account for van der Waals interactions [40]. A supercell model was constructed by superimposing an 11 × 11 MoS_2_ unit cell on a 12 × 12 Au(111) unit cell, with a vacuum layer of at least 10 Å in the z-direction to minimize interactions between periodic images. DFT calculations were performed for each defect model to obtain the charge density distributions, which were then used to simulate STM images using the Tersoff–Hamann approach [41]. In STM simulations, the tip positioning is emulated by placing it at 1.5 above surface sulfur atoms. The STM image simulation follows the standard formalism:

(3) $$\rho_{\mathrm{topo}}\left(r,V\right)=\int_{E_{F}}^{E_{F}+V}D\left(E,r\right)\;dE$$

where $\rho_{\mathrm{topo}}$ represents the topographic density at position r, D(E,r) denotes the local density of states (LDOS) at energy *E*, $E_{F}$ is the Fermi level, and *V* the applied bias voltage. This integration of electronic states within the energy window $\left[E_{F},E_{F}+V\right]$ generates the simulated height-dependent tunneling current map.

## 4. Conclusions

We propose a SAM-CNN integrated framework for automated detection and classification of point defects in ${\mathrm{MoS}}_{2}$ STM images. By combining a segmentation-optimized SAM architecture with a compact CNN trained on limited experimental data (198 samples), our approach achieves 95.06% classification accuracy through atomic-scale noise suppression and hierarchical feature learning. This performance surpasses conventional image processing methods like OpenCV while demonstrating three key advantages: (1) effective suppression of substrate-induced artifacts through SAM’s attention mechanisms, (2) small-sample learning capability enabled by CNN’s parameter-efficient design, and (3) cross-material generalizability evidenced by successful extension to ${\mathrm{WS}}_{2}$/${\mathrm{WSe}}_{2}$ systems and oxide surfaces (e.g., oxygen vacancies in ${\mathrm{TiO}}_{2}$ [42,43] and ${\mathrm{CeO}}_{2}$ [44]). The STM–DFT–CNN workflow establishes a universal protocol for high-throughput nanoscale characterization, enabling rapid defect identification across diverse material systems (including graphene and h-BN substrates) through targeted training data adaptation. This methodology not only advances intelligent automation in materials science but also creates new opportunities for defect engineering in emerging quantum materials and catalytic surfaces.

## Data Availability

The data are available at https://github.com/fiftyfive515/defects (accessed on 10 May 2025).
